# Brain atrophy in middle age using magnetic resonance imaging scans from Japan’s health screening programme

**DOI:** 10.1093/braincomms/fcac211

**Published:** 2022-08-22

**Authors:** Susumu Mori, Kengo Onda, Shohei Fujita, Toshiaki Suzuki, Mikimasa Ikeda, Khin Zay Yar Myint, Jun Hikage, Osamu Abe, Hidekazu Tomimoto, Kenichi Oishi, Junichi Taguchi

**Affiliations:** Department of Radiology, Johns Hopkins University, School of Medicine, 330 Traylor Bldg, 217 Rutland Ave, Baltimore, MD 21205, USA; Tokyo Medical and Dental University, 1 Chome-5-45 Yushima, Bunkyo City, Tokyo 113-0034, Japan; Department of Radiology, The University of Tokyo, Graduate School of Medicine, 7-3-1 Hongo, Bunkyo City, Tokyo 113-0033, Japan; Resorttrust.Inc, Engyou Bldg.8F, Roppongi 7-15-14, Minato-ku, Tokyo 106-0032, Japan; Resorttrust.Inc, Engyou Bldg.8F, Roppongi 7-15-14, Minato-ku, Tokyo 106-0032, Japan; Advanced Medical Care Inc., Midtown Tower 6F, Akasaka 9-7-1, Minato-ku, Tokyo 107-6206, Japan; Resorttrust.Inc, Engyou Bldg.8F, Roppongi 7-15-14, Minato-ku, Tokyo 106-0032, Japan; Department of Radiology, The University of Tokyo, Graduate School of Medicine, 7-3-1 Hongo, Bunkyo City, Tokyo 113-0033, Japan; Department of Neurology, Hidekazu Tomimoto, Mie University 2-174, Edobashi, Tsu, Mie 514-0001, Japan; Department of Radiology, Johns Hopkins University, School of Medicine, 330 Traylor Bldg, 217 Rutland Ave, Baltimore, MD 21205, USA; Tokyo Midtown Clinic, 9-7-1-6F Akasaka, Minato, Tokyo 107-6206, Japan

**Keywords:** brain, MRI, atrophy, dementia, modifiable

## Abstract

Although health screening plays a key role in the management of chronic diseases associated with lifestyle choices, brain health is not generally monitored, remaining a black box prior to the manifestation of clinical symptoms. Japan is unique in this regard, as brain MRI scans have been widely performed for more than two decades as part of Brain Dock, a comprehensive health screening programme. A vast number of stored images (well over a million) of longitudinal scans and extensive health data are available, offering a valuable resource for investigating the prevalence of various types of brain-related health conditions occurring throughout adulthood.

In this paper, we report on the findings of our preliminary quantitative analysis of T_1_-weighted MRIs of the brain obtained from 13 980 subjects from three participating sites during the period 2015–19. We applied automated segmentation analysis and observed age-dependent volume loss of various brain structures. We subsequently investigated the effects of scan protocols and the feasibility of calibration for pooling the data. Last, the degree of brain atrophy was correlated with four known risk factors of dementia; blood glucose level, hypertension, obesity, and alcohol consumption.

In this initial analysis, we identified brain ventricular volume as an effective marker of age-dependent brain atrophy, being highly sensitive to ageing and evidencing strong robustness against protocol variability. We established the normal range of ventricular volumes at each age, which is an essential first step for establishing criteria used to interpret data obtained for individual participants. We identified a subgroup of individuals at midlife with ventricles that substantially exceeded the average size. The correlation studies revealed that all four risk factors were associated with greater ventricular volumes at midlife, some of which reached highly significant sizes.

This study demonstrates the feasibility of conducting a large-scale quantitative analysis of existing Brain Dock data in Japan. It will importantly guide future efforts to investigate the prevalence of large ventricles at midlife and the potential reduction of this prevalence, and hence of dementia risk, through lifestyle changes.

## Teaser

Mori *et al*. investigated midlife brain atrophy and its association with lifestyle using a large MRI data repository in Japan. Ventricular volumes were highly sensitive to ageing and remain robust against protocol variability. The study found significant correlations between ventricular volumes and four known modifiable risk factors of dementia.

## Graphical Abstract

**Graphical Abstract:**
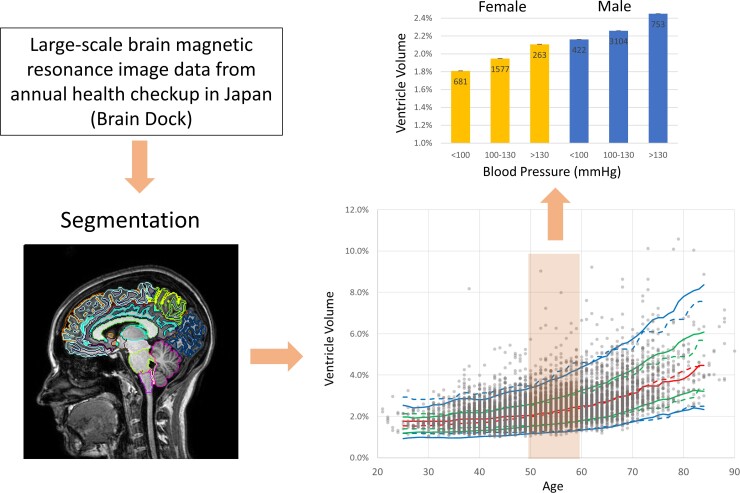

## Introduction

Chronic diseases related to lifestyle choices are becoming a major public health problem. The World Health Organization (WHO) estimated that 61% of all deaths and 49% of the global burden of disease were attributable to chronic diseases. Preventive strategies, notably health screening, plays a major role in identifying populations at risk during pre-symptomatic phases and early interventions. Strikingly, the brain, which is arguably the most important organ in the body, has not been a target of screening, effectively remaining a black box prior to the manifestation of a problem, by which time the problem is often too late to cure. Our brains are physically and chemically well isolated from the environment, and there are no simple and cost-efficient ways of examining their health status. MRI is likely the best available option for evaluating anatomical alterations of the brain, but has rarely been adopted as a health screening because of its high cost.

In this context, Japan is in a unique position. The number of MRI scanners per capita in Japan far exceeds these numbers in the member countries of the Organization of Economic Co-operation and Development, and it is not uncommon for brain MRIs to be performed as a part of Japan’s preventive health screening programme, Brain Dock. This situation allows for a rare glimpse of brain health throughout adulthood (from the ages of 20 to 90 years). The sheer volume of these data could extend to millions of images, as there are many sites that offer more than 10 years of annual follow-up data. Numerous MRI studies have reported age-dependent changes in the brain using MRIs (for example see review by Fox *et al*.^1^). However, their sample sizes rarely reached 1000 or entailed a longitudinal period exceeding 5 years. The availability of a large longitudinal dataset would enable an investigation to address interesting questions, such as how the ageing process takes place within different parts of the brain, their averages and variances, numbers of outliers, and how they are related to lifestyles and long-term brain health.

Utilization of the Brain Dock data is not, however, straightforward for the following reasons. First, the data are distributed across many sites. Second, the scan protocols are heterogeneous because of the absence of nation-wide standardization. Third, the scans are typically of low resolution, with a limited number of available contrasts. Historically, the scans were designed to detect anatomical abnormalities requiring immediate attention, such as neoplasms, aneurysms, inflammation, or evidence of strokes, and not for research on the brain’s anatomy and functions. Therefore, an assessment of the data quality and an evaluation of the feasibility of quantitative analyses are essential for fully exploiting the potential of these data.

Our long-term goal is to evaluate the potential implications of using these MRI data for early detection of populations at risk for dementia through the quantification of brain atrophy. According to the WHO, around 50 million people are currently suffering from dementia worldwide, and this number is expected to increase to 150 million by 2050. Previous studies’ findings have led to a consensus that many forms of dementia are chronic diseases that develop over a decade, with potentially 30–50% of the causes attributed to modifiable lifestyle-associated factors.^2–11^ According to the WHO and the Alzheimer’s Disease Association, these factors include lack of exercise, smoking, excessive use of alcohol, obesity, an unhealthy diet, midlife hypertension, and high blood sugar levels. Additional risk factors include depression, low educational attainment, social isolation, and cognitive inactivity. As the healthcare costs of ageing populations are increasing at unsustainable rates in many countries, identifying opportunities for the prevention of dementia through the management of these modifiable risk factors is becoming increasingly urgent.

There are two key factors pertaining to the effective prevention of lifestyle-related chronic diseases. First, given that the application of preventive measures to the entire population is unrealistic in terms of cost and efficiency, specific high-risk subgroups should be targeted. Second, it is widely believed that such measures are more effective if they are applied during the early phase of a disease. These two points are not specific to dementia and can be applied to all types of chronic diseases, which is precisely the purpose of population-wide health screening.

In this preliminary study, we tested whether the prevalence of brain atrophy in midlife could be characterized using T_1_-weighted images recorded under the Brain Dock programme in Japan. We subsequently examined relationships between the degree of brain atrophy and four known risk factors of dementia: midlife hypertension, high blood glucose levels, obesity, and alcohol consumption.

## Materials and methods

### Site information

The study was performed using data obtained from the Midtown Clinic in Tokyo, Japan. The study was approved by the institutional review board of Midtown Clinic, Japan (#2021-08). The MRI scans were performed at three different sites using 3 T SIEMENS scanners. A total of five different protocols, adopting Siemens’ 3 T MRI 3D-Volumetric Interpolated Brain Examination sequences, were used with different image resolutions as shown in Table 1.

**Table 1:** Sample size and demographic information on the participants for each protocol

	Sample size	Women/men	Ages (average ± std)	Scanner type	Matrix size/voxel size (mm)
Site 1: Protocol 1	2781	976 (1197)/1245 (1584)^a^	53.7 ± 11.2 (F), 53.2 ± 10.8 (M)	Skyra/3T	320 × 320 × 120–128/0.78 × 0.78 × 1.2
Protocol 2	1497	473 (598)/742 (899)	54.5 ± 10.9 (F), 54.2 ± 10.2 (M)	Skyra/3T	320 × 320 × 72–80/0.78 × 0.78 × 2.0
Site 2: Protocol 1	2847	882 (1041)/1495 (1806)	54.2 ± 11.5 (F), 54.0 ± 10.6 (M)	Biograph mMR/3T	640 × 640 × 120/0.39 × 0.39 × 1.2
Protocol 2	3543	1007 (1268)/1036 (2275)	54.8 ± 11.0 (F), 54.4 ± 10.5 (M)	Biograph mMR/3T	640 × 640 × 72/0.39 × 0.39 × 2.0
Site 3: Protocol 1	11 739	3324 (4.609)/4669 (7130)	50.7 ± 11.5 (F), 50.0 ± 10.4 (M)	Spectra/3T	320 × 320 × 64/0.69 × 0.69 × 2.5

^a^ Numbers in parentheses denote the number of scans, which exceeded the number of subjects because multiple scans were performed.

### Subject population

We used data from subjects who received MRI scans between July 2015 and December 2019 in this study. The sample sizes and demographic information of the participants are presented in Table 1. We applied scan data without inclusion or exclusion criteria. Because we used the available data retrospectively without randomization, there could be potential bias in recruitments across scanning sites and/or imaging protocols. The participants received the MRI scans as an option to their routine annual checkup, often partially covered by their employers as a benefit. As a result, the population could be biased to wealthier populations in Japan. Because of the high-quality image inspections performed during the scans, no cases with severe motion artefacts were found. A total of 5105 subjects underwent the mini-mental state examination test, with subjects in their forties, fifties, and sixties, respectively, attaining average scores of 29.0, 29.0, and 28.9. The participants underwent blood tests, blood pressure measurements, and abdominal computerized tomographies (CTs) to measure body fat (visceral fat area at the navel), and they completed a questionnaire about their drinking habits.

### Automated brain segmentation for volume measurements

Automated segmentation was performed using Mvision (Corporate M, Tokyo, Japan), which was based on the multi-atlas pipeline implemented in MriCloud (Johns Hopkins University, Baltimore, MD, USA).^12–16^ As described elsewhere, this pipeline segments the entire brain into five hierarchical structural levels, with the highest (coarsest) level comprising eight structures and the lowest (finest) level comprising 287 structures.^13,14,17–19^ The total number of defined structures across the five levels was 505. The raw volume numbers were converted into relative volumes by dividing the raw volume of each defined structure by the total volume (*TotalVol*), which was calculated by summing all structures, including the brain, sulci, and cranial space volumes.

To evaluate the potential bias introduced by the normalization of the structure volumes based on the proportional approach, residuals method was also tested, in which adjusted volumes (*Vol_adj_*) was obtained from:

$$Vol{-}_{adj}=Vol\;\text{–}\;b(TotalVol\;\text{–}\;TotalVol_{mean})$$

Here, *Vol* is the raw volume of a segmented structure, *b* is the slope from the linear regression of *Vol* and *TotalVol*, and *TotalVol_mean_* is the mean of the total volumes across all subjects.^20,21^

### Data processing and statistical analysis

#### Correlation analysis of volume as a function of the subject’s age

For the analysis of correlations of brain structures with age, normalized volumes were first converted to a log scale to improve homoscedasticity, and polynomial fitting was performed. To examine the influence of the polynomial orders, we used the GridSearchCV package in Python 3.6, which generated 2nd–5th orders depending on the structures. The results were compared with 2nd and 3rd order polynomial fittings, and the effects on correlation coefficients were minimal. The results for the 2nd-order polynomial were reported. For this analysis, we used high-resolution data with the one (first) data point for each subject (obtained using Protocol 1 at the first and second sites; *n* = 4598).

#### Evaluation of the impacts of different protocols

We performed an ANOVA to evaluate the impact of differences in protocols on volume measurements. To compare effect sizes, differences relating to protocol, sex, and age, and their interactions, were analyzed with a three-way ANOVA. To determine age effects, we divided the entire sample into eight decade-based age groups (twenties to nineties). The statsmodels library of Python 3.6 was used for the calculation.

#### Calibration of ventricular volumes across different image resolutions

We tested the performance of the calibration to reduce the effects of image resolution on the measurements of ventricular volumes. Because protocol effects cannot be completely removed, the potential contribution of protocols was compared with the contributions of age and sex. Reducing protocol effects has two important consequences. First, data from various sources can be pooled, thus greatly increasing the sample size. Second, it enables the interpretation of future data by enabling their comparison with existing data in the database, even if the imaging protocols used are not exactly the same. For example, for a given structure volume, sex, and age, it can be determined whether the volume is within the normal range after proper calibration. This is an essential step for measuring brain atrophy as a practice to be adopted within Brain Dock. Evidently, a core assumption is that the image resolution is the single most influential factor among the protocol parameters. As described in the ‘Results’ section, this assumption was tested for the cortical and ventricular volumes.

The calibrations of data with different image resolutions entailed *z*-score conversion. First, the raw volumes were converted to the normalized volume ratios in a log scale as described above. Second, for each age, average and standard deviations of the volumes were separately calculated for each protocol type. For this calculation, data for a ±2-year range (a total of 5 years) were combined to enhance the signal-to-noise ratio. The calculation was performed for the age range 30–75 years. Third, using average and standard deviation values, a 2nd-order polynomial fitting was performed for the age–volume relationship independently for data with scan resolutions of 1.2, 2, and 2.5 mm, respectively. This procedure yielded the mean volume $\bar{V}$(*age*)*^resolution^* and standard deviation *V*_*std*_(*age*)*^resolution^* as a function of age for each resolution. Fourth, using the 1.2 mm data as a reference, we applied the following equation for the data calibration between 1.2 and 2.0 mm and between 1.2 and 2.5 mm:

$$\begin{array}{rl} V(age{)}_{cor}^{2.0\mathrm{mm}}= & \left[V(age{)}^{2.0\mathrm{mm}}-\bar{V}(age{)}^{2.0\mathrm{mm}}\right]\\ & *[V_{std}(age{)}^{1.2\mathrm{mm}}/V_{std}\;(age{)}^{2.0\mathrm{mm}}]\\ & +\bar{V}\;(age{)}^{1.2\mathrm{mm}}, \end{array}$$

where *V*(*age*) and *V*(*age*)*_cor_* denote the volume values for each subject before and after the correction. This approach assumed that the normal distributions of the populations scanned under different imaging protocols were identical.

For the confirmation of the calibration, the age–volume relationships of the data with the three different protocols were separately fitted to 3rd-order polynomial lines before and after the calibration. The fitting was performed by the Seaborn package of Python 3.6.

#### Characterization of population distribution of brain atrophy

Statistical characterization of measurement outcomes relative to population data is a key objective of health examinations. We applied two approaches for estimating the quantile information of each subject: data-driven gradient boosting regression and normal-distribution modelling. For both models, the raw volumes were converted to normalized volume ratios in a log scale, as described above.

Based on the normal-distribution model, $\bar{V}$(*age*)^1.2mm^ and standard deviation *V_std_*(*age*)^1.2mm^ were used to calculate *z*-scores. Given concerns regarding the non-normal distribution of ventricle volumes, gradient boosting regression was also performed to estimate quantiles of volumes of interest for each age group. For this calculation, the GradientBoostingRegression function in the scikit-learn package of Python 3.6 was used with the following parameters: number of estimators = 50, maximum depth = 2, learning rate = 0.1, the minimum number of samples of a leaf = 50, and the minimum number of samples for split = 100.

#### Regression analysis for correlating ventricular volumes with clinical data

In this study, ventricular volumes were correlated with measures of four known risk factors: fasting blood glucose level (mg/dl), maximum blood pressure level (mmHg), frequency of alcohol consumption (times per week), and abdominal fat areas measured with a CT scan. The data at three different image resolutions were first calibrated and pooled. To improve the normality of the distributions, the ventricular volumes and blood glucose levels, areas of abdominal fat, and blood pressure values were first converted to a logarithmic scale. The effect of age was minimized by first binning the data to each decade and subsequently performing independent analyses for the following age groups: 40–49 years, 50–59 years, and 60–69 years. The age-dependency of both independent variables (ventricular volumes) and dependent variables (clinical measures) were further reduced by using the $\bar{V}$(*age*) and *V_std_*(*age*) functions obtained above and fitting out the age response. The multivariate robust linear regression was performed using statmodels in Python 3.6, in which the clinical measures, protocols, and repeated measurements were used as independent variables. The *P*-values associated with the determined slopes were reported.

### Data availability

The approved IRB protocol allows sharing of population-aggregated data, which are available on request from the corresponding author.

## Results

### Effects of age

Table 2 presents a summary of the correlations of structures with age evidencing high correlation coefficients. Information on the constituent substructures of the total cortex and ventricular volumes is also shown. The volume of the entire telencephalon decreased by 1.8% over a 30-year period with *R*^2^ of 0.30. The entire cortex showed a greater degree of change (−4.3%/30 years) with a similar level of age correlation (*R*^2^ = 0.32). The significant volume loss of the telencephalon was attributed to the loss of cortical volumes, with the correlation of the total white matter to age being much smaller (*R*^2^ = 0.05, 30-year change = –1.3%). Within the cortex, the frontal lobes evidenced the highest degree of atrophy and the strongest correlation with age correlation, followed by the parietal and the temporal lobes. The occipital lobe and the limbic system demonstrated a weak correlation with age. Ventricular volumes evidenced one of the strongest correlations with age (*R*^2^ = 0.34) with substantial changes occurring over 30 years (+51.7%). The anterior portions of the lateral ventricles, including the anterior horn and the body of the lateral ventricle (hereinafter referred to as the anterior lateral ventricles) showed the greatest correlation with age and change over 30 years, which coincided with the volume loss in the frontal lobes. Other structures evidencing strong correlations with age included the Sylvian fissure and the periventricular white matter low-intensity areas. The latter comprised white matter areas around the anterior horn of the lateral ventricles with low intensity in the T_1_-weighted images. The expansion of these areas often results from pathological substrates, such as extracellular fluid content, gliosis, disorganized axonal fibres, and demyelination, often referred to as white matter lesions and typically found near the ventricles.

**Table 2:** Brain structures evidencing strong correlations with age among the subjects

	Correlation (*R*^2^)	Change (30–60 years)	Size at 30 years
Telencephalon	0.297 (0.293/0.300)^b^	−1.76% (−1.72/−1.80)	39.6% (39.7/39.4)
Cortex	0.320 (0.323/0.317)	−4.35% (−4.34/−4.35)	20.9% (20.8/21.1)
Frontal lobe	0.298 (0.292/0.304)	−7.80% (−7.60/−8.00)	6.98% (6.89/7.06)
Parietal lobe	0.090 (0.107/0.073)	−4.74% (−4.94/−4.54)	3.72% (3.67/3.77)
Temporal lobe	0.115 (0.117/0.114)	−2.10% (−1.89/−2.30)	5.30% (5.28/5.32)
Occipital lobe	0.005 (0.006/0.003)	−1.99% (−2.33/−1.64)	2.68% (2.75/2.60)
Limbic Cortex	0.002 (0.002/0.002)	−0.10% (−1.13/0.88)	1.74% (1.65/1.82)
Ventricles	0.321 (0.318/0.324)	+59.8% (63.0/56.7)	0.63% (0.66/0.61)
Anterior ventricles	0.308 (0.307/0.309)	+68.6% (70.9/66.3)	0.44% (0.46/0.41)
Posterior ventricles	0.298 (0.293/0.302)	+54.7% (56.8/52.6)	0.13% (0.14/0.12)
Inferior ventricles	0.203 (0.220/0.185)	+14.6% (18.8/10.3)	0.07% (0.06/0.08)
Sylvian fissure	0.371 (0.368/0.374)	+20.3% (19.5/21.2)	0.35% (0.37/0.33)
Periventicular WM-LI area^a^	0.410 (0.419/0.401)	+48.5% (62.2/34.8)	0.03% (0.02/0.04)

^a^ WM-LI denotes white matter low-intensity area.

^b^ The numbers in the parentheses are from left and right hemispheres.

### Characterization of volume distributions of the normal population

**Figure 1:**
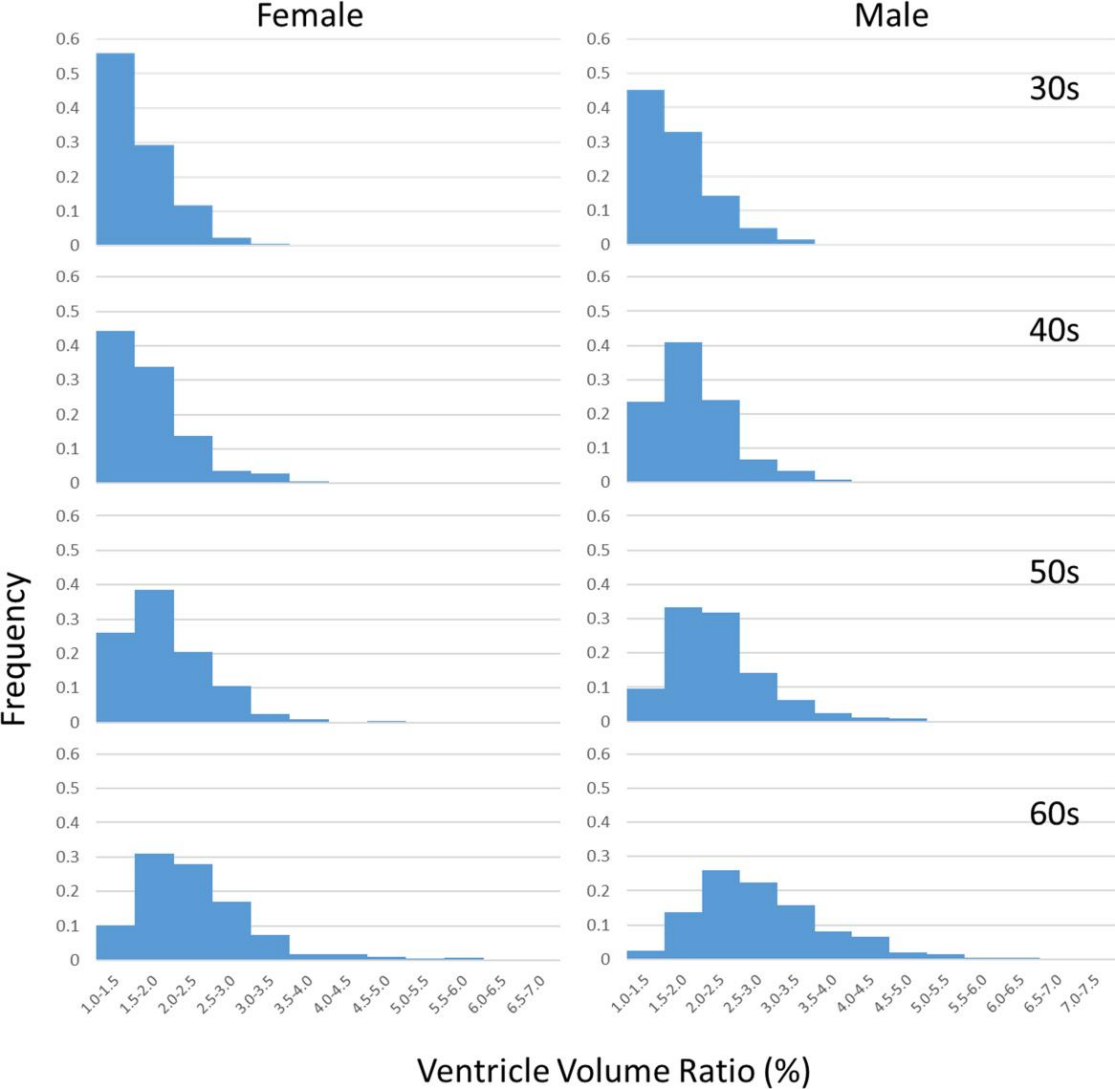
**Histograms of ventricular volumes for each decade using a linear scale**. The *x*-axis shows the normalized ventricular volume (% relative to all structures combined) and the *y*-axis shows the frequency.

Figure 1 depicts histograms of ventricular volumes for each decade obtained using high-resolution data (Protocol 1, Sites 1 and 2, *n* = 5628). Several notable features of brain atrophy were observed. First, a consistent shift in the distribution towards larger volumes was apparent due to ageing. Second, the distributions were strongly skewed towards the larger ventricular volumes. Third, a portion of the population showed impressive resilience against atrophy (<1.5% ventricular volume) even in their sixties.

**Figure 2:**
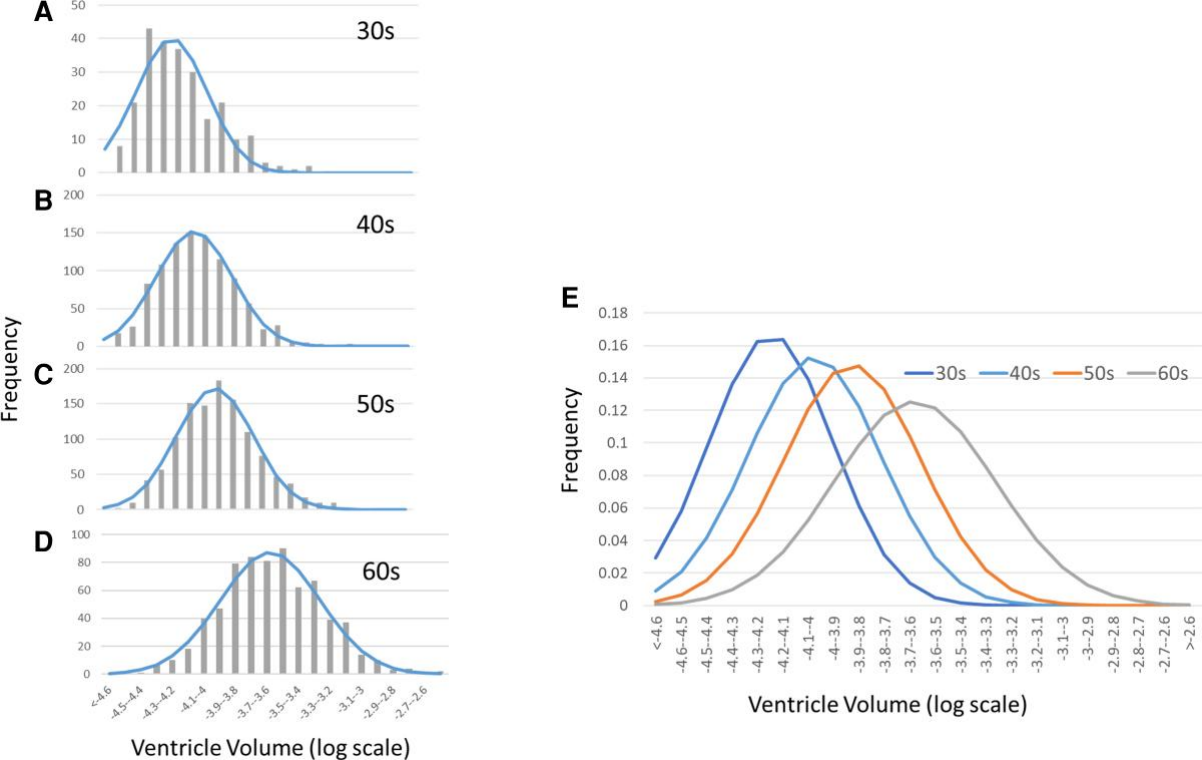
**Distributions of ventricular volumes within a logarithmic scale and fitting to the normal-distribution model**. (**A**)–(**D**) The actual histograms and results of fitting to normal distributions for the thirties, forties, fifties, and sixties age groups, respectively. Data from male subjects are shown for demonstration purposes. (**E**) The fitted normal-distribution curves over four decades.

As shown in Fig. 2A–D, the distributions of the ventricular volumes more closely approximated the normal distributions after conversion to a logarithmic scale, although they were still skewed towards higher volumes (data shown were from Protocol 1, Sites 1 and 2). Assuming normal distributions, the population distributions can be modelled using averages and standard deviations, as shown in Fig. 2E. This type of population characterization is an essential first step for interpreting data obtained from individuals.

### Effects of protocols on the segmentation results

#### Ventricle volumes

**Figure 3:**
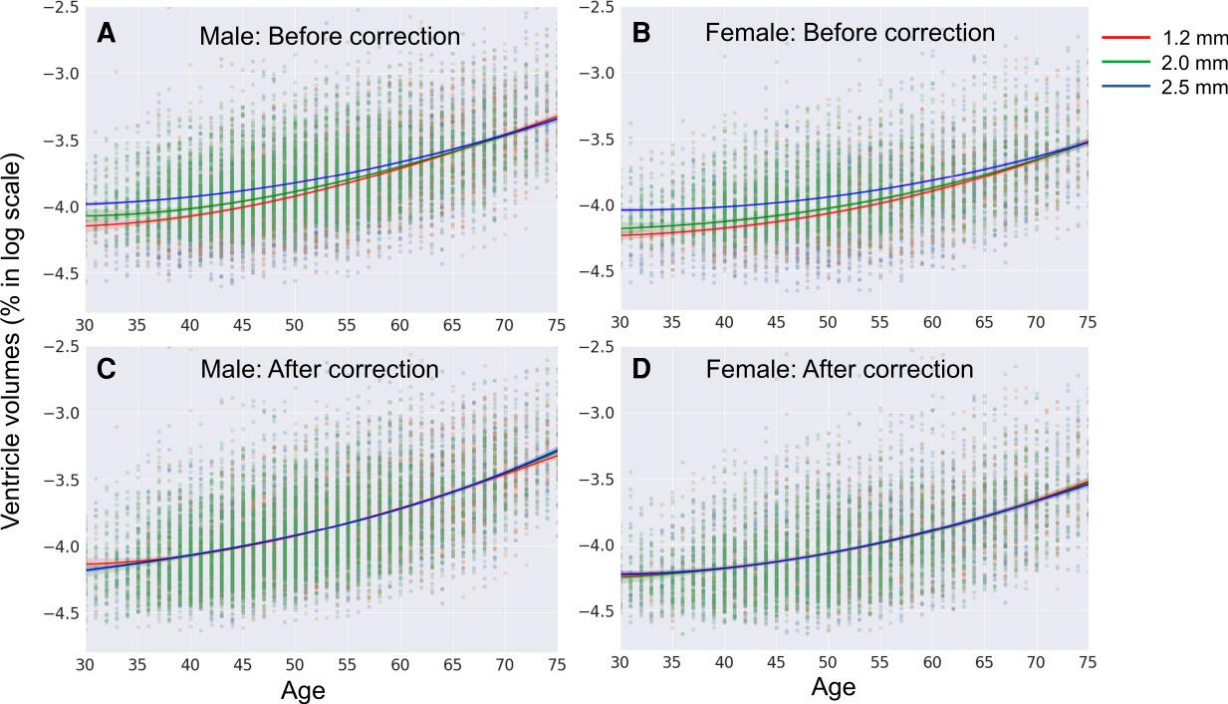
**Scatter plots for age–volume relationships before and after calibration with three different scan resolutions**. (**A** and **B**) Ventricular volumes from raw data in a logarithmic scale as a function of age for male (**A**) and female (**B**). (**C** and **D**) Ventricular volumes obtained after calibration using data with 1.2 mm slice thickness as a reference. The fitted curves are 3rd-order polynomial lines with 95% confidence intervals. After the calibration, the difference among the three protocols was non-significant (*P* = 0.923), based on the *F*-values of three-way ANOVA.

An ANOVA was performed for data with the same resolution obtained from two different sites. The *P*-value between the two sites at 1.2 mm (Site 1: Protocol 1 versus Site 2: Protocol 1) was 0.094. Similarly, the *P*-value between the two sites at 2.0 mm (Site 1: Protocol 2 versus Site 2: Protocol 2) was 0.125. The interactions of the protocols with two dominant biological effects (age and sex) did not reach statistical significance (*P* > 0.436). Therefore, data with the same slice resolutions were pooled for the subsequent analysis. The effect of the protocol across the three different slice thicknesses (1.2 versus 2.0 versus 2.5 mm) was highly significant (*P* < 0.001). The amount of its contribution to the total variance was 1.15%, approaching ¼ of the sex effect (4.12%), while the age effect had the largest contribution (30.2%). These results can be clearly appreciated in Fig. 3A and B, in which the log ventricle volumes of the male and female participants were plotted against the subjects’ ages. The differences were more evident for younger subjects whose ventricle sizes were smaller, whereas they shrank with an increase in ventricular size. These differences were most likely due to increased partial volume effects in images with lower resolutions. This finding suggests that lower resolution images have less power to differentiate ventricular size for subjects with smaller ventricles and are therefore not ideal for characterizing age-dependency for younger populations.

#### Cortical volumes

The data from the two sites with the same slice thickness (Site 1 versus Site 2 with 1.2 or 2 mm slice thickness) were statistically different (*P* < 0.0001) unlike the ventricular volumes. Therefore, we were unable to pool the data from two sites where the same slice resolution was applied. The effect of protocol differences was much larger than that obtained for ventricular sizes; the effect size (contribution to the total variance) of the protocol difference (23.1%) was larger than that of sex (2.1%) and age (15.7%). The cortical volumes tended to be overestimated at lower resolutions for all ages, although the loss of cortical volumes occurred for all protocols at similar rates. This finding suggests that data from different protocols could not be easily calibrated and pooled across the five protocols. Homogenization of protocols assumes more importance than ventricle-based analyses for data combined from multiple sites. Alternatively, each site would need to create its own database for interpreting cortical volume data.

Given non-significant differences in the volumes of ventricles with the same slice thickness obtained using different scanners, we decided to perform calibration across studies that used different image resolutions. Figure 3C and D shows ventricular volumes as a function of subject age after calibration. These plotted volumes indicate that the calibration method could effectively reduce the effect of image resolution. The three-way ANOVA performed for three different resolutions showed non-significant differences (*P* = 0.923).

#### Investigation of brain atrophy using ventricles

In this preliminary study, we focused on ventricular size as an index of age-dependent loss of brain tissues because they have a high level of correlation with age. Moreover, compared with the cortex, which also shows a high correlation with age, ventricle volumes are more robust against variabilities in image protocols. Furthermore, we deemed cross-protocol calibration based on image resolution to be feasible.

**Figure 4:**
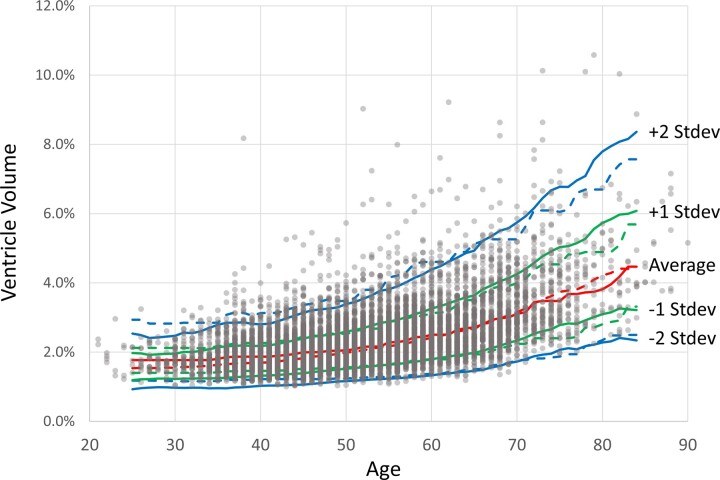
**Estimation of *z*-scores of ventricular size based on the normal-distribution model and gradient boosting regression**. Estimation of *z*-scores (or equivalent percentiles) of ventricular size based on the normal-distribution model (solid lines) and gradient boosting regression (dotted lines) for the pooled data.

Applying the above-described calibration approach and a normal-distribution model in a logarithmic scale, we characterized the age-dependent distribution using data pooled from five different protocols (Fig. 4). Accordingly, it became possible to estimate the *z*-score of each subject, which is one of the ultimate goals of the Brain Dock programme.

Figure 4 reveals that the number of subjects with substantially larger ventricles appeared to increase among those in their late thirties and above, which is expected from the non-normal distribution observed in Fig. 2. Although careful interpretation is needed, as the sample size also increased for subjects towards the forties and fifties, it is reasonable to assume that large ventricles in the subjects who have passed their late thirties (*z*-score > 2.0) are acquired and not inherited traits.

Given potential deviation from the normal distribution, population percentiles equivalent to *z*-scores of 1 (15.9%) and 2 (2.3%) were calculated using a gradient boosting regressor (Fig. 4, dotted line). Larger discrepancies from the normal-distribution model were observed among subjects below 40 years and above 70 years as the sample size decreased towards the edge of the age distribution. In areas with fewer data, the model-based estimation (i.e. the normal-distribution model) may be more robust. For the 40–60 year age group, slight effects of the skewed distributions were observed: the *z*-score = ±2.0 lines from the normal-distribution model were slightly lower than the data-driven estimation obtained with the gradient boosting regression.

#### Correlation with clinical and lifestyle data

**Figure 5:**
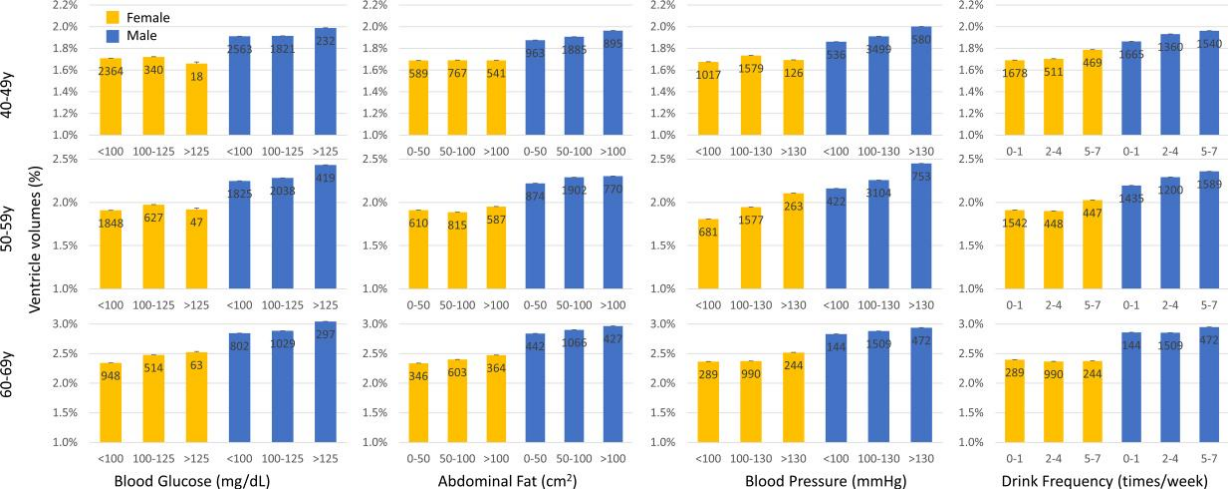
**Relationship between four potential risk factors and ventricular volumes**. For each analysis, the populations were binned according to the extent of risk factors, and their averaged ventricular volumes were presented. The error bars are standard errors and the numbers inside the bars are sample sizes.

**Figure 6:**
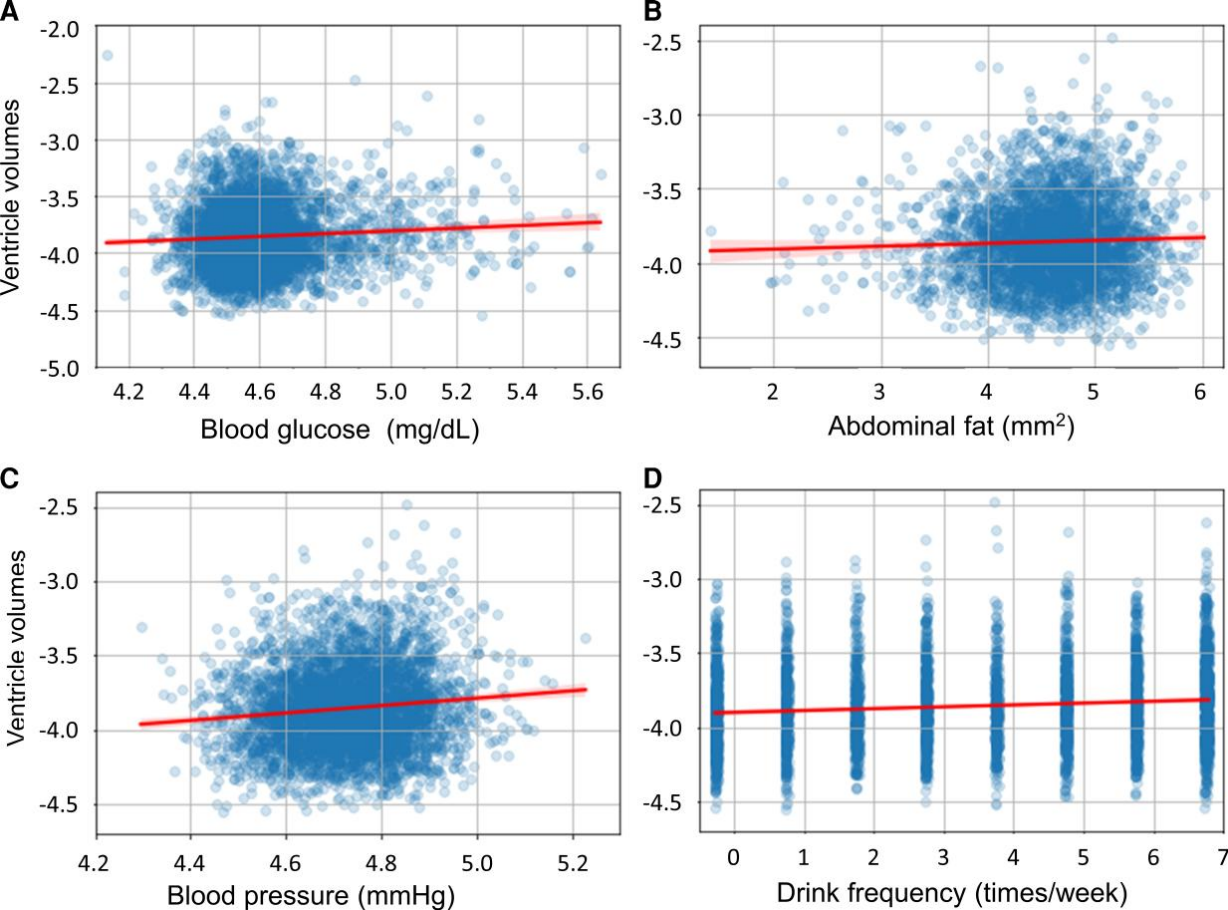
**Representative scatter plots with linear regression results**. Results are from male subjects in their 50s. (**A**) Blood glucose levels. (**B**) Abdominal fat areas. (**C**) Blood pressure levels. (**D**) Drinking frequencies. The lines were based on robust linear regressions and the ranges (shaded areas) indicate 99% confidence intervals. The multivariate linear regression was used for the fitting with *P*-values of 2.87E−4 (**A**), 0.0234 (**B**), 7.88E−5 (**C**), and 4.66E−12 (**D**).

The results of the linear regression analysis are shown in Table 3. In Table 3, *P*-values <0.05 are indicated by underlines and those <0.0125 (Bonferroni corrected for four independent measurements) by boldface for visual clues. Figure 5 visually depicts data that were binned according to the degree of the clinical and lifestyle data. The larger ventricular volume was significantly related to higher fasting blood glucose levels in men who were in their fifties and sixties (*P* < 0.0008). For areas of abdominal fat, the correlations with ventricular volume were weaker than that of blood glucose levels, but male populations in their forties and fifties showed moderately low *P*-values (*P* < 0.05). Clearer correlations were found for blood pressure levels and drinking frequencies. High levels of *P*-values (*P* < 0.001) were found in male populations in their forties and fifties with hypertension and in female populations in their fifties. *P*-values were low (*P* < 0.001) for drinking frequency for both men and women in their forties and fifties. Figure 6 shows four representative plots for the linear regiression results from male subjects in their 50s.

**Table 3:** Results of linear regression analyses of ventricular volumes and four risk factors

			*n*	*R* ^2^	*P*−value^a^	Slope
Blood glucose level	40	Female	2719	0	0.973 (0.852)	−9.00E−05
		Male	4614	0.001	0.130 (0.0179)	3.00E−04
	50	Female	2518	0	0.987 (0.0681)	3.00E−04
		Male	4282	0.005	**2.87E−4 (4.59E−8)**	1.00E−03
	60	Female	1518	0.001	0.0172 (0.0289)	7.00E−04
		Male	2128	0.005	**7.27E−4 (6.47E−4)**	9.00E−04
Abdominal fat	40	Female	1896	0	0.874 (0.817)	5.80E−03
		Male	3743	0.002	0.0405 (0.0845)	2.19E−02
	50	Female	2010	0	0.927 (0.171)	8.40E−02
		Male	3543	0.001	0.0234 (0.0313)	1.93E−02
	60	Female	1313	0	0.694 (0.654)	7.70E−03
		Male	1935	0	0.573 (0.528)	3.90E−03
Blood pressure	40	Female	2722	0.001	0.228 (0.0156)	7.00E−04
		Male	4615	0.007	**5.26E−4 (2.79E−5)**	1.70E−03
	50	Female	2521	0.016	**4.42E−4 (7.64E−10)**	2.50E−03
		Male	4279	0.011	**7.88E−5 (4.72E−11)**	2.20E−03
	60	Female	1523	0.001	0.0645 (0.0648)	8.00E−04
		Male	2125	0.003	**0.0113 (0.00419)**	1.30E−03
Frequency of alcohol consumption	40	Female	2659	0.006	**8.00E−6 (1.70E−4)**	8.20E−03
		Male	4570	0.04	**3.02E−4 (4.63E−4)**	6.20E−03
	50	Female	2438	0.004	**3.55E−4 (7.90E−4)**	6.70E−03
		Male	4224	0.013	**4.66E−15 (1.21E−12)**	1.24E−02
	60	Female	1465	0.001	0.215 (0.330)	4.00E−03
		Male	2105	0.003	0.0138 (0.0224)	6.30E−03

^a^ Values inside the parentheses are based on brain size normalization using residuals method. For visual clues, values below 0.05 are underlined and below 0.0125 (Bonferroni corrections with four independent measurements) are shown by bold fonts.

To test the effect of potential bias introduced by the brain size normalization based on the propotional approach, we tested regression using *Vol_adj_* values and the re-calculated *P-*values were provided in Table 3 (values inside the parentheses). We found the results from two different types of normalization approaches gave similar results.

For those with significant correlations, the *R*^2^ values (ratio of contributions of each factor to the total variance) remained within a range of 0.3–1.7%. These small effect sizes are expected given the large number of potential risk factors that can affect brain atrophy, indicating the need for large sample sizes for their characterization.

Overall, younger female populations did not show high levels of significance. This result could be attributed to experimental conditions in addition to biological contributions. First, the sample size of women was relatively smaller than that of men. Second, as shown in Fig. 5, only a small proportion of the population had higher (worse) blood glucose levels and blood pressure, leading to a small dynamic range that was less suitable for correlation analysis. Third, in this study, young female populations had the smallest ventricular size. As shown in Fig. 3, the low-resolution data had less power to differentiate ventricular sizes within this population; the lowest boundary for ventricular size that can be differentiated using the 2.5 mm slice thickness is about –4.1 in a logarithmic scale (1.67% of the total brain volume). This issue would impede an investigation of the correlations between ventricular sizes and clinical data for populations with small ventricles.

## Discussion

### Potential of large-scale brain MRI study

Whereas previous large-scale, MRI-based brain ageing studies covering the entire span of adulthood are very rare, this situation has shifted in recent years, with the initiation of large projects funded by federal governments. These studies usually have a clear focus in terms of diseases, populations, or biological questions. For example, more than US$200 million has been invested in the Alzheimer’s Disease Neuroimaging Initiative, which was initiated in 2004 with 800 elderly subjects and was expanded to cover 2000 subjects, entailing repeated scans.^22^ The Adolescent Brain Cognitive Development Study, which targeted paediatric populations, generated more than 10 000 images.^23^ More recently, the Biobank Project in the UK, which has received more than US$70 million in funds since 2015, has begun collecting brain MRI scans for subjects aged between 40 and 69 years, with a long-term target of providing longitudinal scans for up to 100 000 subjects. As of December, 2020, 43 000 scans have been generated, of which 1400 have been scanned twice.^24,25^

The scarcity is due to the high costs and efforts required, especially for longitudinal follow-ups. In this respect, Japanese Brain Dock data are unique. There are over 1000 sites providing MRI services in Japan. Numerous sites have been operating for more than 15 years, with many participants, aged 20–90 years, returning after 1–2 years. The sheer amount and duration of available data could easily eclipse research-based studies. However, these data entail several disadvantages compared with well-organized research studies. First, the demography of the participants is skewed towards wealthier individuals, who can afford costly scans or who have health benefits that include MRI scans. Second, the scan protocols are not standardized, and many scans are based on typical clinical scan protocols that tend to use thicker slices.

Despite these limitations, an examination the potential implications of these data is merited. The scientific question pertains to whether biologically or clinically important information about the brain ageing process, pathological ageing, and relationships with clinical or lifestyle data can be extracted. From a more engineering-oriented perspective, the knowledge accumulated from these past data could be useful for managing population-level brain health in the future.

### Using ventricles as a marker of the brain atrophy

A core question relating to the characterization of brain ageing concerns its location. The maximum amount of information on location that can be extracted from one image is for each voxel. More than 1 million voxels in a brain are apparent at a 1 × 1 × 1 mm resolution and theoretically, the age-dependence of brain atrophy at the level each voxel can be examined, which is the purpose of voxel-based morphometry.^26^ However, the vast amount of locational information obtained for each subject quickly becomes a statistical burden with an increase in the sample size and the initiation of correlation studies with non-image parameters. To apply brain MRI data for complex correlation analyses practically, the location dimension evidently needs to be reduced. By defining 505 structures using a segmentation tool, the locational information dimension would be reduced more than 1000-fold. However, a list of 505 volumes is probably still too large to handle in an evaluation of brain health.

In this study, our dimension-reduction strategy was based on two factors. First, we hypothesized that structures evidencing high age dependency would be good candidates for monitoring brain ageing. Although there are some conflicting reports, regional variability of age-dependent volume loss, shown in Table 2, is in agreement with the majority of previous publications. For example, our results showed that the cortex had a higher degree of age-related volume loss, whereas white matter was preserved to a greater extent.^27–29^ Within the cortex, the frontal lobes have widely been identified as the region with the earliest onset of atrophy,^29–33^ whereas preservation of the limbic system, including the hippocampus, has also been reported.^28,29,32,33^ Age-dependent increase of ventricular size was uniformly observed across all publications.

The second dimension-reduction strategy focused on robustness against differences in protocol. We found that ventricular volumes satisfied these conditions and therefore used them to characterize atrophy progression due to ageing. However, this choice does not preclude the possibility that there are other structures that could be more suitable as markers for monitoring brain health. For example, it is possible that a certain pathological condition would affect a specific structure and, thus, measurements of such a structure could provide a better indicator of the disease (e.g. cerebellar volumes in ataxia patients). On the other hand, a recent study has shown that the size of lateral ventricles could be a sensitive marker of brain functions.^34^ Clearly, more research is needed to enable further exploitation of the data.

The robustness of the measurement of ventricular volume was probably attributable to the clear contrast between the tissue and cerebrospinal fluid, which was stable across protocol differences. Conversely, the boundary between grey and white matter was more susceptible to variability, and even if the resolution was the same, the data derived from different scanners led to significant differences. This is to be expected given the high sensitivity of tissue contrasts relating to various scan parameters, including flip angles and pulse timing. Currently, the vast majority of available MRI scans in the Japanese Brain Dock system have slice thicknesses above 2 mm. Our findings indicated that ventricular volume could be a good initial candidate for investigating the prevalence of brain atrophy using available data. In the future, it would be important to use more homogenized high-resolution T_1_-weighted images to support a more detailed analysis of various brain tissue structures. For example, various applications of data science technology have been postulated in recently publications for the dimension reduction through the usage of feature extraction tools, in which features from voxels of the entire brain or a large number of segmented structural units were used.^35–37^

### Prevalence of brain atrophy in middle-age populations

After calibrating and pooling all of the data, we characterized average and standard deviations of the ventricular volume for each age group (Fig. 4). This was likely the most important achievement of this study. The Brain Dock programme is designed to identify populations with potentially or highly abnormal results and recommend further disease-targeted evaluations for these groups. Establishment of a normal range of ventricular volumes for each age enables evidence-based decision making tailored for each individual who avails of the MRI service, as in the case of various lab tests, such as blood and urine tests that are components of health checkups. Our results indicate that MR images can be quantitatively interpreted in a similar manner. Of course, abnormal values do not always indicate clinically adverse findings. For example, known anatomical variations occur in the cerebrospinal fluid space and are regarded as clinically insignificant (e.g. cavum septum pellucidi, cavum vergae, and mega cisterna magna). Therefore, further research is required for clinical interpretation. Nonetheless, it is reasonable to assume that accelerated ventricular enlargement is not ideal. We found a notable increase in populations with ventricles that considerably exceeded two standard deviations who were at least in their late thirties. Because we did not observe these data points in younger populations, we assumed that they were mostly acquired rather than innate features.

At the same time, a large number of subjects showed impressive resilience against ageing. Ventricular size in 11% of the population in their sixties was less than the average size of those in their thirties. Future studies should attempt to identify modifiable risk factors that contribute to not only the accelerated progression of ventricular enlargement, but also to a brain anatomy that is young in appearance.

Our findings aligned with recent publications about estimation of brain ages from features in T_1_-weighted images.^38,39^ Once the age–volume relationship of a structure of interest is obtained, the age can be estimated from the volume. The discrepancy between the estimated and real ages could be used as an indication of the brain health. Our approach used a single structure for the analysis. Recent publications have reported more advanced approaches, in which multiple structures were integrated using machine learning tools.^35,40^ These approaches have been extended not only to evaluate brain ageing processes but also to diagnose neurological diseases.

### Correlation with four types of clinical and lifestyle data

The correlation between modifiable risk factors and brain atrophy assumes importance of this study, as it implies that addressing these factors could change the course of brain deterioration. Previous publications have reported that relationships exist between brain atrophy and diabetes, hypertension, obesity, and alcohol consumption.^41–44^ However, to the best of our knowledge, this is the first study reporting on the simultaneous evaluation of all four factors using the same dataset with such a large sample. Of these factors, we found that hypertension and the frequency of alcohol consumption had the greater impacts on ventricular enlargement. The contributions of these factors to the total variance were, at most, 1–2% (*R*^2^ values in Table 3). Strikingly, this finding coincides with previous estimations of risk factors of Alzheimer's disease in which hypertension (2%), more than 21 units of alcohol consumption weekly (1%), and obesity (1%) were identified as risk factors at midlife and diabetes (1%) in later life.^45^

In our study, the significant differences tended to occur in populations in their fifties, which could be due to several reasons. First, this group comprised the largest population in our study sample. Second, the effects of lifestyles are expected to be cumulative over time. However, as various types of lifestyle-related diseases start to set in, the quality of clinical and lifestyle data also deteriorates; blood glucose and blood pressure may be managed with drugs, and body weight and alcohol consumption may decrease due to illness. As subjects age, their past histories encompassing clinical and lifestyle information become more critical for accurate analyses. This could be the reason why correlations were not stronger in their sixties than fifties in this cross-sectional study.

There are numerous publications reporting on the relationships between brain volume loss and modifiable risk factors. For example, midlife obesity has been linked to lower brain volumes, which is associated with cognitive decline^46^ and dementia.^47,48^ The Biobank study, conducted in the UK, is probably the largest study on population health that includes brain MRI scans. In light of its findings, Dekkers *et al*.^49^ reported a significant negative correlation between obesity and subcortical grey matter volumes. High blood glucose levels have been associated with brain volume loss^50^ and low cognitive performance.^51,52^ Likewise, correlations between brain volume and hypertension have been reported.^53,54^ A relatively large-scale study (*n* = 550) reported that alcohol consumption led to both brain volume loss and cognitive decline over a 30-year period.^55^ The frontal lobes have been identified as the brain structure most susceptible to deterioration,^56^ while the medial temporal lobes are also affected in older populations.^57^ Studies have also reported recovery of brain volumes after periods of abstinence (for a review, see Kril *et al*.^44^ and Garavan *et al*.^58^). The findings of these studies suggest that early detection of populations at risk (with an increased atrophy rate revealed in MRI scans) and interventions entailing lifestyle changes could have beneficial impacts, which is the precise aim of Brain Dock.

### Brain MRI for studying dementia

Recent studies have shown that dementia is attributable to modifiable risk factors related to life.^2–10^ In addition, there is increasing evidence from epidemiological, clinical, imaging, and biomarker studies indicating that cognitive impairment induced by Alzheimer’s disease may be a clinically silent disorder that begins in midlife, with its terminal phase characterized by dementia.^9,59–62^ Interventions that target individuals at midlife to address health-related behavioural changes around modifiable risk factors may reduce individuals’ risk of developing dementia in later life and, as a consequence, the prevalence of dementia.^5,8,63^ The prevention of dementia through the early detection of populations at risk and the enforcement of healthier lifestyles would be key strategies for population-level health management. However, the link between the appearance of any specific biomarker in asymptomatic individuals and the subsequent emergence of clinical symptoms remains unclear.^10,60^ If such a link can be established, it will open up a crucial window of opportunity for interventions targeting modifiable risk factors.^60^

Brain MRI is one of the modalities that can be applied for directly observing the brain anatomy, and consequently, directly measuring parameters relating to brain atrophy and white matter lesions. There is an abundant literature describing relationships between atrophy of certain brain regions and occurrences of dementia, such as Alzheimer’s disease. The hippocampal atrophy is one of the key anatomical features of Alzheimer’s disease. Many studies have also reported dementia-associated atrophy in various brain regions, including other limbic structures (amygdala and entorhinal cortex), temporal lobe structures, grey matter nuclei, and cortical grey matter. However, these findings, which were derived from comparison of patients with dementia and age-matched control groups, inevitably focusing on late-life (typically with an average age of 70 years) populations. It is unclear whether such late-life features could also be an effective marker for early detection of dementia at midlife.

In this study, we demonstrated the feasibility of quantitatively identifying people with statistically large ventricles at each age. We further demonstrated that the emergence of large ventricles was apparent in participants from their late thirties onward. What is particularly concerning was the non-normal distributions that was skewed towards larger sizes, potentially indicating emergence of pathological cases. Further, the volumes had significant correlations with known risk factors for dementia. These risk factors are acknowledged to cause brain volume loss at midlife and to be associated with the onset of dementia. In light of these findings, we can assume that there is an association between populations with significantly large ventricles and the risk of dementia. However, we were unable to determine the cause-and-effect relationship in this cross-sectional study. To do so would require a prospective longitudinal study of a large population for several decades. Another potentially compounding factor was idiopathic normal pressure hydrocephalus that often occurs in late-life and induces ventricular enlargement.^11^ Nonetheless, the extensive brain MRI database in Japan could be an important resource for future research aimed at investigating whether early intervention through lifestyle changes would lead to the reduction of accelerated brain atrophy as well as dementia risk.

## Conclusion

We performed a feasibility analysis to characterize midlife brain atrophy using Japanese health checkup data. There exists a large MRI data repository in Japan encompassing all stages of adulthood, which would be ideal resource for performing a large-scale epidemiological study of midlife brain atrophy and its association with lifestyle. We found that ventricular volumes are highly sensitive to ageing and remain robust against protocol variability. Using them as a marker, we examined correlations with four known modifiable risk factors of accelerated brain atrophy and dementia: high blood glucose level, hypertension, obesity, and alcohol consumption. We found significant correlations existing between ventricular volumes and these risk factors. This study reveals the promising potential of using existing health checkup data in Japan to manage brain health conditions, including dementia.

## Competing interest

S.M. is one of the co-founders of AnatomyWorks and Corporate M. S.M. is CEO and K.O. is a consultant of AnatomyWorks. These arrangements are being managed by the Johns Hopkins University in accordance with its conflict of interest policies.

## Abbreviations

WHO =: World Health Organization
WM-LI =: white matter low intensity
